# Relational similarity in wild bumblebees: the role of spatial alignment complexity

**DOI:** 10.1007/s10071-025-02012-6

**Published:** 2025-11-17

**Authors:** Gema Martin-Ordas

**Affiliations:** Division of Psychology, University of Stirling, Stirling, UK

**Keywords:** Relational similarity, Mapping strategies, Reasoning, Invertebrates, Bumblebees

## Abstract

Being able to abstract relations of similarity is considered one of the hallmarks of human cognition. Importantly, previous research has shown that other animals—both vertebrates (e.g., primates) and invertebrates (e.g., bees)— are capable of spontaneously attending to relational similarity in spatial mapping tasks. These tasks require individuals to find a reward in an array of, for example, three objects, after observing a reward being hidden in a different array of three objects. Studies with primates have shown that performance in this type of task is influenced by the distribution of the objects in the arrays. Here I investigated whether wild bumblebees’ relational abilities are also affected by the spatial complexity of the arrays (i.e., three horizontally aligned stimuli). In Experiment 1, bees were presented with two arrays separately: in one condition, the arrays were placed next to each other (forming a line) and in the other, the arrays were placed in two different rows. In Experiment 2, the two arrays were also placed in two rows, but the rows were misaligned. Bees succeeded in both Experiments and in the three different distributions of the arrays. The results suggest that bees were comparing the two arrays and recognized the common relational features in both arrays. Studies like the ones presented here highlight the importance of studying social insects to understand the evolution of cognition.

**Supplementary Information:**

The online version contains supplementary material available at 10.1007/s10071-025-02012-6.

## Introduction

Analogical reasoning is the ability to perceive and use relational similarity between different situations (Gentner 2003; Penn et al. 2008). This type of reasoning is argued to be critical for abstract thinking and is usually believed to be a rather unique feature of human cognition (e.g., Gentner 2003; Gentner and Hoyos 2017). Generally, establishing an analogy involves mapping relational features from a well-known situation to a more “unfamiliar” situation (Gentner and Rattermann 1991). For example, when answering “duck is to duckling as tiger to?” we should notice the relation parent–offspring in the well-known situation (i.e., duck and duckling) to, then, map it to our target situation (i.e., tiger and?) and correctly answer “cub.” Thus, understanding that critical object properties are not necessarily the properties of the objects individually, but the relations of the properties of the different objects to each other is fundamental for recognizing relational similarity—being this the critical aspect of analogical reasoning (e.g., Haun and Call 2009).

Using match-to-sample tasks (MTS), in which a sample stimulus is presented with two comparison stimuli, a correct and an incorrect match, vertebrates (e.g., primates and birds; Christie et al. 2016; Smirnova et al. 2015) and invertebrates (e.g., bees; Giurfa et al. 2001) have been shown to recognise relational similarity. However, there are some methodological limitations with this paradigm. The MTS paradigm entails large amount of training and performance can be explained by simply accounting for the perceptual variability between the presented stimuli—rather than by identifying the relation between the stimuli (Fagot et al. 2001). Work with vertebrates has overcome these issues by using small-scale spatial mapping tasks (e.g., Christie et al. 2016; Hribar et al. 2011) and recent studies have also done so with insects (Martin-Ordas 2022, 2023). Typically, in spatial mapping tasks, subjects see, for example, 3 boxes placed next to each other (*Baited array*), and they watch an experimenter placing a reward in one of these 3 boxes (left, middle, or right). Then, subjects are presented with a second set of 3 boxes (*Searching array*), and they are asked to find the corresponding reward in this Searching array. Recognizing the similarities in the spatial organization between the two arrays is fundamental for individuals to succeed (e.g., Haun and Call 2009).

In non-human animals (henceforth, animals), successful performance in spatial mapping tasks has been shown to be influenced by factors like previous experience with similar but easier problems (Haun et al. 2006), proximity between the objects in the *Searching* and *Baited* arrays (Haun and Call 2009) or the positioning of the arrays (Hribar et al. 2011). For example, Hribar et al. (2011) presented apes with a Baited array and a Searching array (each array was integrated by 3 cups) that were placed one behind the other (i.e., forming two rows), next to each other (i.e., forming a line) or one behind the other in two misaligned rows. To succeed, apes had to find a reward in the Searching array, after observing a reward being hidden in the Baited array. Their results showed that great apes’ best performance was when the two arrays were one behind another. However, apes struggled when the arrays were forming a line. Interestingly, the authors also reported that apes did not seem to map the spatial relations between the objects of the two arrays (e.g., left cup of the Baited array with the left cup of the Searching array); rather, apes appeared to make choices based on the use of shared landmarks between the Baited and Searching arrays (e.g., the edge of the surface where the cups were placed). This strategy was also shown to be used by young children in the same task (Hribar et al. 2012).

Based on their results, Hribar and colleagues (2011, 2012) suggest that two strategies can be at play in this type of spatial matching tasks. Individuals could either be mapping by using the objects in the arrays (i.e., so-called *aligned strategy*) or by using objects outside the arrays (i.e., so-called *landmark strategy*). When using the aligned strategy, individuals are mapping locations based on two relations (e.g., the middle cup is right of the left cup and *left* of the right cup). In contrast, when using the landmark strategy mapping is possible by using only one relation (e.g., the cup is next to an edge). Thus, these two strategies require different number of relations to be established. Of course, this distinction is important for predicting performance in spatial mapping tasks. As indicated above, when the reward is hidden on the left or right cup, subjects only have to consider one spatial relation. Consequently, it would be expected that individuals do not find it difficult to find the reward in the Searching array when the reward was hidden in these locations. In contrast, when the reward is hidden in the middle cup, the aligned mapping strategy is based on two relations. Not surprisingly, and given their complexity, midpoint mapping strategies emerge later than single-relation mapping strategies in development (DeLoache and Brown 1983; Quinn et al. 2003; Simms and Gentner 2019; Uttal et al. 2006). Research has also shown that overall animals struggle in tasks that require the encoding of a midpoint relationship (e.g., fish: Sovrano et al. 2007; primates: Jones et al. 2002; Potì et al. 2010). Importantly, in their relational mapping study, Hribar et al. (2011) also found that apes’ performance was worse when the middle cup was baited compared to when the left or right cups were baited and argued that this could have been due to having to encode two spatial relationships.

Being able to successfully forage in a complex environment involves establishing relationships between different objects (e.g., the purple flower is *next to* the yellow flower). Therefore, it is conceivable that establishing relational similarities between different events, contexts or objects is an adaptive strategy for bees. In laboratory contexts, bees have been shown evidence of relational processing—acquiring and extrapolating implicit knowledge- and relational learning—learning same/different, larger/smaller rules- (e.g., honeybees: Giurfa et al. 2001; Avarguès-Weber et al. 2020; Howard et al. 2017; bumblebees: Brown and Sayde 2013). Recent studies with wild bumblebees have shown that bees can spontaneously use spatial mapping abilities (Martin-Ordas 2022, 2023). For example, in Martin-Ordas (2022) bumblebees were presented with two sets of two objects each (Baited and Searching arrays). Bees experienced that only one object of the Baited array was dipped in sucrose. Their task was to find the corresponding strip in the Searching array. In the first experiment, the objects in both arrays looked the same but their alignment was manipulated. In the second experiment, the objects were different so that spatial matches competed with object matches. The results showed that in both experiments wild bumblebees spontaneously attended to relational similarity between the arrays.

**Fig. 1:**
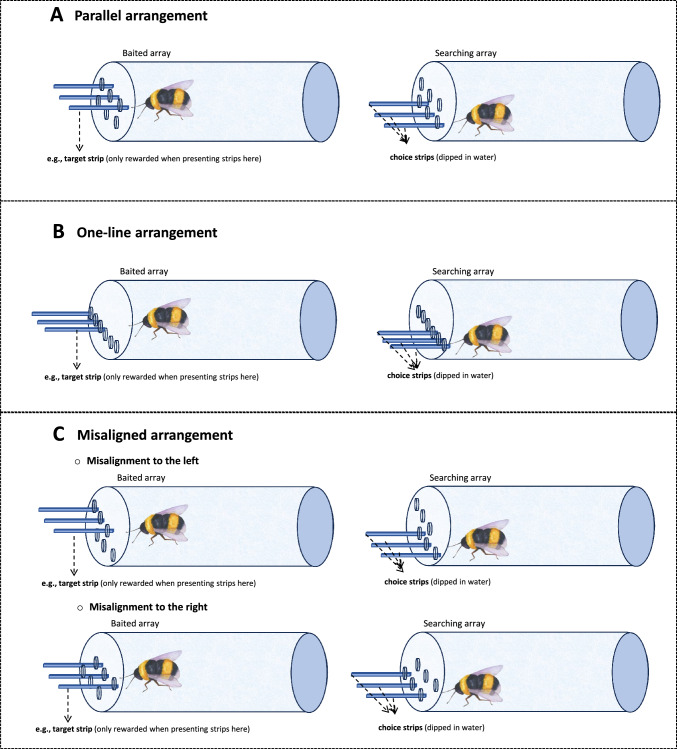
Representation of the strips’ alignments for Experiment 1: (**A**)*, Parallel arrangement and* (**B**) *One-line arrangement* and Experiment 2:* (C) Misaligned arrangement*. Bees faced three objects and experienced one of them dipped in sucrose (Baited array). Bees’ task was to search among the objects when presented a second time (Searching array). For each Experiment, the position of the target strip is counterbalanced across trials

The main objective of the current studies was to further explore wild bumblebees’ relational abilities in a spatial mapping task that allowed directly comparing bees’ performance with that in previous studies with children (Hribar et al. 2012) and great apes (Hribar et al. 2011). This approach allows identifying similarities and differences in the flexible use of mapping strategies as well as preferences in mapping strategies that might be shared across a wide range of species. Following Hribar et al.’s work, the constellation of the two arrays of strips was manipulated. Specifically, the Baited and Searching arrays were either placed next to each other, forming a line, or were aligned perfectly one below the other (Experiment 1) or were placed one below the other but in a misaligned manner (Experiment 2; see Fig. 1). If bees rely on a mapping strategy when making their choices in the Searching array, they will succeed in both experiments. Importantly, if the complexity of the arrays influences bumblebees’ abilities to establish these mapping strategies, bees’ performance is expected to be better when both arrays are spatially aligned (Experiment 1) compared to when they are presented in one line (Experiment 1) or in a misaligned manner (Experiment 2). In the current studies, bumblebees were also presented with two 3-strips arrays—rather than 2-strip arrays, as previously done in Martin-Ordas (2022). If bees make their choices in the Searching array using the landmark strategy, they will be expected to find selecting particular locations (e.g., middle) more challenging than selecting others (e.g., left, right).

## Experiment 1: parallel vs one-line stimuli arrangement

Bees were presented with two conditions that differed in the positioning of the Baited and Searching arrays. In the *Parallel* and *One-line* conditions, the strips in the Baited and Searching arrays had the same relative position within the array. Thus, if the baited strip in the Baited array was the left strip, then the correct strip in the Searching array was also the left strip. That is, the correct strip matched the relative location to the reward within the previously presented baited array. However, whereas in the *Parallel* condition the Searching array was below the Baited the array, in the *One-line* condition the Searching array was next to the Baited array. Based on previous findings (Hribar et al. 2011), it was expected that positioning the two arrays next to each other would increase the difficulty to recognize the relational commonalities between the arrays compared to when the arrays are placed in parallel.

### Subjects

The data was collected between May and June 2022 in Stirlingshire (UK). A total of 30 bees were captured and they all completed at least 10 out of the 12 trials. The final sample was composed of 30 bees of the following species: *Bombus pascuorum* (n = 16), *Bombus terrestris complex* (n = 6), *Bombus pratorum* (n = 6) and *Bombus hypnorum* (n = 2). Sex was visually identified (females = 28) and no queens were tested. This experiment as well as Experiment 2 received ethical approval from the University of Stirling’s Ethics Committee (Project name: Cognition in wild bees). All methods were performed in accordance with the relevant guidelines and regulations.

### Apparatus

A transparent plastic tube (11 × 4.5 cm) with 2 sets of holes through which the stimuli could be inserted was used (see Fig. 1). For the *Parallel* condition, the top (i.e., Baited array) and bottom set of holes (i.e., Searching array) consisted of 3 holes each with 4 mm between them. The distance between the Baited and Searching arrays was 4 mm. For the *One-line* condition, the Baited and Searching arrays were next to each other. The distance between the two arrays was 4 mm. Blue paper strips (3 × 0.2 cm) were used as stimuli: 3 were introduced through the top set of holes (Baited array) and 3 through the bottom set (Searching array). The strips were fixed in playdoh to introduce them simultaneously in the tube.

### Procedure

Experiments were always conducted between 7:30am and 10am. Subjects were left in the tube on average for 1 h prior to testing to allow them to habituate to the tubes and become motivated to forage (Muth et al. 2017). Bees were caught directly from flowers by using the testing tubes in which the experiments were conducted. This minimized the manipulation of the bees. To have the same environmental elements for all bees, playdoh containers were placed on both sides of the tube.

The procedure was the same for the *Parallel* and *One-line* conditions (Fig. 1A-B). Subjects first were presented with the Baited strips on the top array (*Parallel* condition) or left array (Experimenter’s perspective; *One-line* condition). One of the strips was dipped in 50% (w/w) sucrose and the other two strips were dry. Once the bee made contact with the strip dipped in sucrose—either by using its antennae or proboscis— it was given (on average) 5–6 s to drink the solution. Bees were allowed to explore the 3 strips so they could notice that only one of them was rewarded. Then, the Baited objects were removed, and the E introduced the Searching strips when the bees were, at least, 1.5–2 cm away from the Searching Array. These strips were dipped in water. A choice was considered when the bees touched the strip with the antennae or proboscis. Each bee received a total of 12 trials and the position of the reward—left, middle or right— was counterbalanced across trials. The inter-trial-intervals were approximately 2 min for each bee. During this time, subjects were allowed to freely move in the tube. New paper strips were used for each trial and in each array. Importantly, bees did not receive any training prior to these trials.

### Analyses

Data was analysed using R version 2025.05.1 + 513 (R Core Team 2021) using a binomial general linear mixed model (GLMM) (Bolker et al. 2009). The dependent variable was bees’ choices. Specifically, for each trial bees’ first selected stimulus was coded as 1 and the non-selected ones as 0. The independent variable was experimental condition (i.e., Parallel = 1 and One-line = 0 conditions) and the location of the rewarded strip (i.e., Middle = 1, Left = 2, Right = 3) as categorical variables. A random factor was the individual bees. Previous findings with similar tasks (Martin-Ordas 2022, 2023; Muth et al. 2017) did not find species differences. However, a second model also including bee species as an independent variable was conducted to analyse if species had any effect in the current experiments (see Supplementary Information). No differences between species were found. A final model was run with bees’ choices as the dependent variable, trial, condition and food location as independent variables and subjects as random factor (see Supplementary Information). Bees’ performance did not decrease with trials. This indicated that bees’ responses were not affected by the lack of reward in the Searching array. Wilcoxon tests were used to analyse if performance was significantly above chance (i.e., 33.33%). To run these analyses, the percentage of correct responses was calculated out of the trials performed for each bee. These individual percentages were then used to compare them against chance levels. P-values below 0.050 were considered to provide evidence for significant differences.

### Results and discussion

**Fig. 2:**
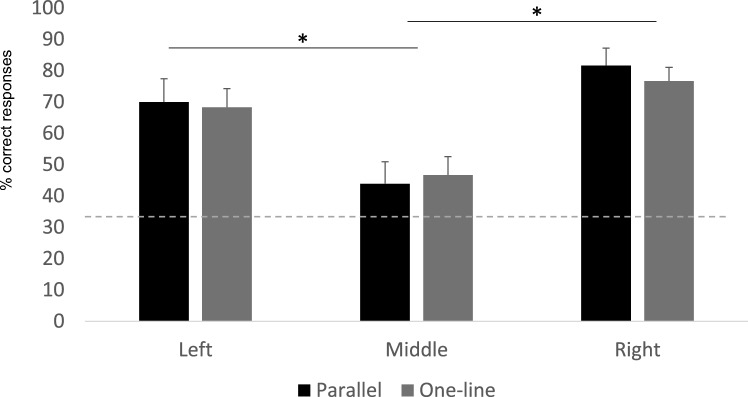
Represents the percentage of correct responses in the *Parallel* and *One-line* conditions of Experiment 1. Note that data here are presented separated for each condition. However, for the analyses the conditions were grouped since there were no differences in bees’ performance for the Parallel and One-line condition. The percentage of correct responses was calculated out of the trials performed for each bee. The individual percentages were then used to calculate the group mean. The asterisks indicate the significant differences for bees’ choices between the different locations of the rewarded strip. The dotted line represents the chance level (33.33%).The bars represent the SEM

No interaction between condition and reward position was found (estimate *SD* = 0.136,* z* = 0.455, *P* = 0.648, 95% CI = 0.441 to 0.713). Therefore, the interaction was removed from the model. Condition did not have an effect in bees’ performance (estimate *SD* = 0.064,* z* = 0.255, *P* = 0.798, 95% CI = −0.448 to 0.133; Fig. 2). Subjects performed significantly above chance in both *Parallel* (Wilcoxon test: *W* = 120, *P* < 0.001) and *One-line* conditions (Wilcoxon test: *W* = 120, *P* < 0.001). Importantly, there was a significant effect of the reward position on bees’ choices—with bees performing worse when the reward was in the middle strip compared to the right (estimate *SD* = 1.549,* z* = 5.197, *P* < 0.001, 95% CI = 0.97 to 2.15; Fig. 2) and left strip (estimate *SD* = 0.989,* z* = 3.594, *P* < 0.001, 95% CI = 0.45 to 1.53; Fig. 2). Subjects found the reward significantly above chance in the three potential locations (Wilcoxon test; Left: *W* = 429, *P* < 0.001; Middle: *W* = 374, *P* = 0.003; Right: *W* = 465, *P* < 0.001).

Contrary to the predictions, the positioning of the two arrays did not influence bees’ performance. Bees successfully found the correct strip in both the *Parallel* and *One-line* conditions. While it is true that bees could be choosing the closest strip, for example, in the *Parallel* condition when the rewarded strip is on the left or the right, this explanation cannot account for bees’ relatively worse performance when the reward is in the middle strip, regardless of the condition. This “proximity” explanation cannot account for bees’ performance in the *One-line* condition either. This is because bees did not choose the closest strip to the Baited array— irrespective of the position of the rewarded strip in the Baited array. Thus, it is possible that bees did use the information from the Baited array to infer the correct strip’s position in the Searching array. To confirm that bees are reliably using a spatial matching strategy (i.e., aligned strategy), in Experiment 2 bees were presented with two misaligned arrays in which alternative strategies (e.g., proximity-based) compete with the aligned strategy and lead to the selection of different strips.

## Experiment 2: misaligned stimuli arrangement

**Fig. 3:**
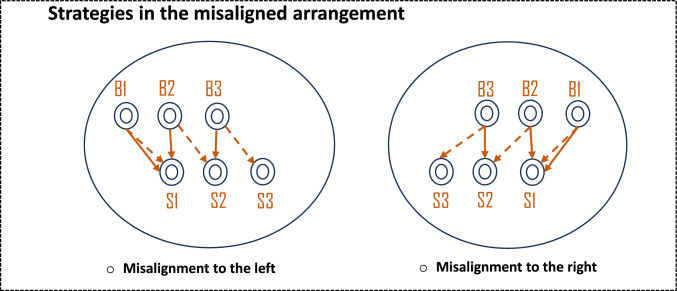
Frontal view of the apparatus of Experiment 2. The dashed arrows represent which strip bees would choose if they use the relational similarity between strips (spatial relation strategy). The solid arrows represent the strip that bees would choose if they used the proximity strategy. When bees found the nectar in strip B1 could use either strategy. When bees found the nectar in B2 and B3, bees should use a spatial relation strategy to succeed

In this Experiment, the Baited and Searching arrays were now positioned in two misaligned rows. Specifically, the middle strip in the Baited array was positioned above the right or the left strip in the Searching array (Figs. 1C and 3). This arrangement allowed examining whether bees would show a preference for a relational strategy when the use of competing, and simpler strategies is also possible. Additionally, the misaligned stimuli arrangement facilitated further investigating bees’ difficulties to select the correct strip when the rewarded strip was found in the middle.

### Subjects

The data was collected between June and July 2022 in Stirlingshire (UK). A total of 30 bees were captured, and 22 bees completed all the trials. The rest completed, at least, 9 trials. The final sample was composed of 30 bees of the following species: *Bombus terrestris complex* (n = 14), *Bombus pascuorum* (n = 12), *Bombus pratorum* (n = 2) and *Bombus hypnorum* (n = 2). Sex was visually identified (females = 28), and no queens were tested.

### Apparatus

As in Experiment 1, a transparent plastic tube (11 × 4.5 cm) with 2 sets of holes (see Fig. 1C) was used. In this case, the sets of holes were misaligned—such that the centre strip in the Baited array was aligned either with the right or left strip in the Searching array (see Fig. 3). As in Experiment 1, blue paper strips (3 × 0.2 cm) were used as stimuli.

### Procedure

The same procedure as for Experiment 1 was followed. Bees first were presented with the Baited strips on the top array (Baited array). As in Experiment 1, one of the strips dipped in sucrose and the other two were dry. Once the bee explored the 3 strips, the Baited objects were removed, and the E introduced the Searching strips. For 50% of the subjects, the Baited array was misaligned to the left (Experimenter’s perspective) and for the other 50% to the right (Experimenter’s perspective; see Fig. 1C). Each bee received a total of 12 trials, and the position of the rewarded strip was counterbalanced across trials.

### Analyses

Data was analysed using R version 2025.05.1 + 513 (R Core Team 2021) using a binomial general linear mixed model (GLMM) (Bolker et al. 2009). A first model was run to examine if the position of the misalignment (towards left or right) had any effect in bees’ performance. The dependent variable was bees’ choices. Specifically, for each trial bees’ first selected stimulus was coded as 1 and the non-selected ones as 0. The independent variable was misalignment position and the reward position (e.g., B1 = 1, B2 = 2, B3 = 3; see Fig. 3) as categorical variables, and a random factor was the individual bees. B1 and B3 refer to the outer strips and B2 to middle strip. Two different spatial strategies that bees could use to make their choices were coded (see Fig. 3): (a) Relational. The two strips have similar spatial relations to the other strips within their respective array; (b) Proximity. The two strips are closer to each other than to any other potential strip in the Searching array. As for Experiment 1, two other models were run: one was conducted to analyse the effect of species, and the second one included trial as an independent variable (see Supplementary Information). The results showed that neither species nor trial had a significant effect in bees’ choices. Wilcoxon tests were used to analyse if performance was significantly above chance (i.e., 33.33%). As in Experiment 1, the percentage of correct responses was calculated out of the trials performed for each bee. These individual percentages were then used to compare them against chance levels. P-values below 0.050 were considered to provide evidence for significant differences.

### Results and discussion

**Fig. 4:**
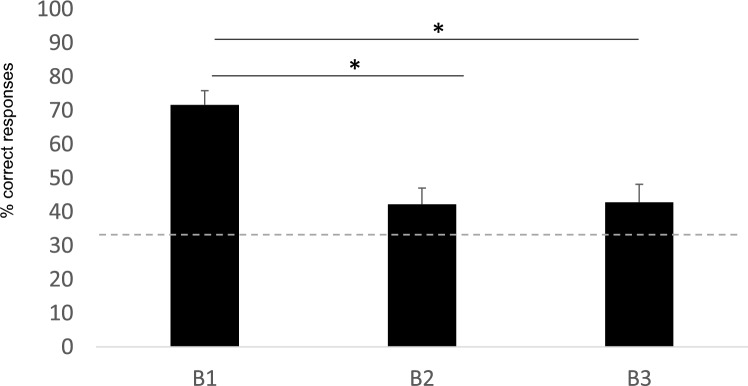
Represents the percentage of correct responses in the Misaligned condition of Experiment 2 (see Figure 3). The percentage of correct responses was calculated out of the trials performed for each bee. The individual percentages were then used to calculate the group mean. The asterisks indicate the significant differences for bees’ choices between the different locations of the rewarded strip. The dotted line represents the chance level (33.33%). The bars represent the SEM

Since the interaction between condition and reward position was not significant (estimate *SD* = −0.09,* z* = −0.352, *P* = 0.724, 95% CI = −0.646 to 0.447), it was removed from the model. There was no effect of condition in bees’ performance (estimate *SD* = 0.337,* z* = 1.507, *P* = 0.1319, 95% CI = −0.100 to 0.777) and subjects selected the correct strip significantly above chance (Wilcoxon test: *W* = 374, *P* < 0.001). However, there was a significant effect of the rewarded position on bees’ choices. Bees performed better when the reward was in the B1 strip compared to the B2 (estimate *SD* = −1.221,* z* = −4.354, *P* < 0.001, 95% CI = −1.781 to −0.679; Fig. 4) and B3 strip (estimate *SD* = −1.160,* z* = −4.168, *P* < 0.001, 95% CI = −0.715 to −0.612; Fig. 4). No differences were found between B2 and B3 (estimate *SD* = 0.061,* z* = 0.231, *P* = 0.817, 95% CI = −0.459 to 0.58).

### Strategies

When the reward was placed in B1, proximity *and* relational strategy led to the correct response, S1, and bees selected this strip significantly above chance (Wilcoxon test: *W* = 432, *P* < 0.001). When the middle strip was baited (B2), the correct response could only be achieved by using the relational strategy and bees did so significantly above chance (Wilcoxon test: *W* = 292, *P* = 0.042). Note that when B2 was baited bees did not select the closest strip (S1) significantly above chance (Wilcoxon test: *W* = 278.5, *P* = 0.345). Finally, when the reward was found in B3, bees used more frequently the relational strategy, although their performance did not reach significant levels (Wilcoxon test: *W* = 297, *P* = 0.084). In this case, the closest strip was not selected significantly above chance either (Wilcoxon test: *W* = 284.5, *P* = 0.147).

Overall, bees consistently selected the correct strip when the Baited and Searching arrays were misaligned. Their performance was best when both a relational and proximity-based strategy could be used (i.e., B1). However, bees also selected the correct strip when the rewarded strip was in the middle; that is, when only a relational strategy could be used (i.e., B2). Finally, when the rewarded strip was B3, bees did not show a clear preference for either strategy. These results indicate that bees use the relational strategy consistently for B1 and B2. However, when B3 is rewarding bees fail to reliably use the relational or proximity strategy to find the correct strip.

## General discussion

Spatial cognitive abilities are of fundamental importance to foraging animal species. Being able to encode the location of an object *in relation to* another object (i.e., *spatial relationships*) is critical for successful foraging (e.g., Burgess et al. 1999; Gentner 2003)*.* In two Experiments bumblebees experienced different array alignments of a small-scale spatial mapping task. In Experiment 1, the Baited and Searching arrays were either placed in two rows or next to each other. In Experiment 2, the arrays were also placed in two rows but misaligned. Bees’ performance was significantly above chance in the three different alignments. The results also showed that bees’ choices were partly determined by the location of the rewarded strips. Whereas in Experiment 1 bees performed better when the rewarded strip was located on the left and right compared to when it was in the middle, in Experiment 2 bees struggled when the reward was found in one of the outer strips (i.e., B3).

The current task setup allows to start disentangling what strategy bees might be using when selecting a strip in the Searching array. As mentioned before, a selection based on the proximity of the strips between the arrays fails to explain the findings. This is for three reasons. First, in Experiment 1 it was difficult for the bees to find the correct strip when the reward was experienced in the middle strip. Second, in the *One-line condition* bees did not consistently choose the closest strip to the Baited array (i.e., left). Finally, when the rows were misaligned (Experiment 2), bees’ choices were largely consistent with a mapping strategy.

Thus, one could suggest that bees relied on relational reasoning to succeed in the present tasks. To do so, bees would have needed to consider each array as a single unit integrated by 3 objects/strips with specific relations among each other and these relations would have been to be considered from their own perspective. This is important because in a study using a similar relational paradigm, Martin-Ordas (2022) showed that bees prefer using allocentric over egocentric strategies. Moreover, when trained to use both strategies, bees still showed a preference for the use of allocentric strategies, though a small number of bees also used egocentric strategies. This could indicate that, in the present experiments, bees were using external cues to select the correct strip in the Searching array. Studies with insects have shown that to find food resources or their nest bees and ants encode the appearance of the landmark and, later, “match” the stored “snapshot” (i.e., “snapshot matching”) with the current visual input (e.g., Dittmar et al. 2010; Durier et al. 2003; Webb 2019). The present results, however, cannot be explained by the snapshot matching strategy, otherwise bees would have struggled with the *One-line* condition in Experiment 1.

It is true that bumblebees showed a lower performance when the reward was found in the middle strip in Experiment 1, and this could be interpreted as bees’ performance not being consistent with a relational strategy. However, this difficulty was only evident in Experiment 1. Bees did match the middle strips in Experiment 2, which suggests that bees could map these relations when the arrays are misaligned. In addition, Experiment 2 also showed that bees prefer the relational over the proximity strategy.

How could, then, bees’ choices be explained? It is possible that the different array alignments might have led bees to use different strategies in Experiments 1 and 2. Following Hribar et al. (2011, 2012), in Experiment 1, bees might have processed the strips as being independent units within the larger frame of reference of the tube and its surroundings (e.g., external landmarks). That is, bees could have established a relation between the playdoh containers and the location of the strips—rather than among the strips themselves. This is why their performance was better when the reward was found in the outer strips compared to the middle one. Note that the middle strip was not placed near a particular landmark but next to the strips that were next to a landmark. In contrast, bees’ performance in Experiment 2 could be best explained by them using a relational strategy. It is possible that relational strategies offer the advantage of appreciating structural similarities when the arrays have a more complex spatial distribution (e.g., Leech et al. 2008), and it is more challenging to rely on landmarks. For example, one of the outer strips in this spatial alignment, B3, was always placed in the middle of the tube and not associated with any landmark. This could have favoured, then, bees relying on a spatial relational strategy.

An explanation based on the use of different strategies in different tasks, however, could be a less parsimonious account since it relies on the combination of several cognitive abilities. First of all, the consideration of each constellation as having different levels of complexity and bees adapting their performance accordingly. Second, switching between strategies across trials. Note that bees did perform worse when the reward was in the middle compared to when the reward was in the outer strips. However, they still correctly selected the middle strip significantly above chance. Finally, inhibiting the use of a landmark strategy. This is particularly true in Experiment 2 when the reward was found in B1, since this was the strip closest to an external landmark. Additionally, recent research shows that bumblebees may be rather limited when it comes to self-control and inhibitory strategies (Baciadonna et al. 2025).

Previous work has demonstrated that bees can learn to abstract relational representations (i.e., “sameness”) in the context of colours, smells, sizes and quantities (e.g., Giurfa 2021; Howard et al. 2017, 2018). Studies have also shown that bumblebees have extraordinary spatial memory skills (e.g., Heinrich 1979; Ohashi et al. 2008) and that they spontaneously use relational reasoning in small-scale spatial mapping tasks (Martin-Ordas 2022, 2023). The results presented here replicate previous findings and extend them by providing evidence that bees flexibly use relational reasoning in different contexts (i.e., spatial alignments). This contrasts with what it has been previously found in great apes (e.g., Hribar et al. 2011). It is true that the paradigms presented here were intended for testing wild bees. Thus, certain factors such individual experience could not be controlled for, and it might have affected bees’ performance in these tasks. Future research with laboratory bees could help to shed light on whether individual learning experience plays an important role in this type of reasoning paradigms.

Relational reasoning plays a critical role in many human cognitive abilities—from problem solving to inferential reasoning or language acquisition (e.g., Gentner 2003). Relational reasoning is also argued to facilitate adaptation to complex and changing situations (Wasserman et al. 2017). Certainly, foraging is a challenging activity for bumblebees (Chittka and Thomson 2001). In addition to making decisions considering the quality and quantity of the rewards, when foraging bees also face a changing landscape given the ephemerality of the resources (Baracchi 2019). Since foraging has been argued to drive the emergence of complex cognitive abilities in bees (e.g., Chittka and Thomson 2001; Hills 2006), it is conceivable, then, that spatial mapping might have also developed in this context. This would also suggest that bees’ spatial cognitive abilities are not as simple as previously believed (e.g., Collett and Collett 2002; Menzel et al. 2005). However, the tasks used in the current experiments are small-scale spatial tasks—which might not have triggered the spatial skills required when foraging. Future research could investigate bees’ relational reasoning abilities in free-flying foraging tasks. This would allow assessing this cognitive skill in contexts with higher ecological validity.

In conclusion, bumblebees displayed relational matching strategies. Their performance varied across the different alignments, which suggests that the spatial distribution of the arrays plays an important role bees’ reasoning strategies. Studies like the ones presented here indicate that research with social insects is very useful to investigate the evolution of cognition, in general, and what factors are at play in cognition in insects, in particular.

## Supplementary Information

Below is the link to the electronic supplementary material.

Data and script for Experiment 1 (12 KB)

Data and script for Experiment 2 (8 KB)

Supplementary information (462 KB)

Data for Experiments 1 and 2 (44 KB)

## Data Availability

All data generated or analysed during this study are included in this published article [and its supplementary information files].

## References

[CR1] Avarguès-Weber A, Finke V, Nagy M, Szabó T, d’Amaro D, Dyer AG et al (2020) Different mechanisms underlie implicit visual statistical learning in honey bees and humans. Proc Natl Acad Sci U S A 117:25923–25934. 10.1073/pnas.1919387117 32989162 10.1073/pnas.1919387117 PMC7568273

[CR2] Baciadonna L, Rovegno E, Bigazzi G et al (2025) Assessing the limits of delay of gratification in bumble bees. Sci Rep 15:24363. 10.1038/s41598-025-08616-9 40628794 10.1038/s41598-025-08616-9 PMC12238607

[CR3] Baracchi D (2019) Cognitive ecology of pollinators and the main determinants of foraging plasticity. Curr Zool 65:421–424 31423133 10.1093/cz/zoz036 PMC6688568

[CR4] Bolker BM, Mollie EB, Connie JC, Shane WG, John RP, Henry MHS, Jada-Simone SW (2009) Generalized linear mixed models: a practical guide for ecology and evolution. Trends Ecol Evol 24:127–135. 10.1016/j.tree.2008.10.008 19185386 10.1016/j.tree.2008.10.008

[CR5] Brown M, Sayde J (2013) Same/different discrimination by bumblebee colonies. Anim Cogn 16:117–125. 10.1007/s10071-012-0557-z 22945434 10.1007/s10071-012-0557-z

[CR6] Burgess N, Jeffery KJ, O’Keefe J (1999) The hippocampal and parietal foundations of spatial cognition. Oxford Univ Press, New York, pp xi, p 490

[CR8] Chittka L, Thomson JD (2001) Cognitive ecology of pollination: animal behaviour and floral evolution. Cambridge University Press

[CR9] Christie S, Gentner D, Call J, Haun DBM (2016) Sensitivity to relational similarity and object similarity in apes and children. Curr Biol 26:531–535. 10.1016/j.cub.2015.12.054 26853364 10.1016/j.cub.2015.12.054

[CR10] Collett T, Collett M (2002) Memory use in insect visual navigation. Nat Rev Neurosci 3:542–552. 10.1038/nrn872 12094210 10.1038/nrn872

[CR11] DeLoache JS, Brown AL (1983) Very young children’s memory for the location of objects in a large-scale environment. Child Dev 54:888–897 6617310

[CR12] Dittmar L, Stürzl W, Baird E, Boeddeker N, Egelhaaf M (2010) Goal seeking in honeybees: matching of optic flow snapshots? J Exp Biol 213:n2913–2923. 10.1242/jeb.043737 10.1242/jeb.043737 20709919

[CR13] Durier V, Graham P, Collett TS (2003) Snapshot memories and landmark guidance in wood ants. Curr Biol 13:1614–1618 13678592 10.1016/j.cub.2003.08.024

[CR14] Fagot J, Wasserman EA, Young ME (2001) Discriminating the relation between relations: the role of entropy in abstract conceptualization by baboons (*Papio papio*) and humans (*Homo sapiens*). J Exp Psychol Anim Behav Process 27:316–328 11676083

[CR15] Gentner D (2003) Why we’re so smart. In: Gentner D, Goldin- Meadow S (eds) Language in mind: advances in the study of language and thought. The MIT Press, Cambridge, pp 195–235

[CR16] Gentner D, Hoyos C (2017) Analogy and abstraction. Top Cogn Sci 9:672–693. 10.1111/tops.12278 28621480 10.1111/tops.12278

[CR17] Gentner D, Rattermann MJ (1991) Language and the career of similarity. In: Gelman SA, Byrnes JP (eds) Perspectives on language and thought. Cambridge University Press, Cambridge, pp 225–277

[CR18] Giurfa M (2021) Learning of sameness/difference relationships by honey bees: performance, strategies and ecological context. Curr Opin Behav Sci 37:1–6 35083374 10.1016/j.cobeha.2020.05.008 PMC8772047

[CR19] Giurfa M, Zhang S, Jenett A, Srinivasan MV (2001) The concepts of ‘sameness’ and ‘difference’ in an insect. Nature 410:930–933. 10.1038/35073582 11309617 10.1038/35073582

[CR20] Haun DBM, Call J (2009) Great apes’ capacities to recognize relational similarity. Cognition 110:147–159 19111286 10.1016/j.cognition.2008.10.012

[CR21] Haun DBM, Rapold CJ, Call J, Janzen G, Levinson SC (2006) Cognitive cladistics and cultural override in hominid spatial cognition. Proc Natl Acad Sci USA 103:17568–17573 17079489 10.1073/pnas.0607999103 PMC1859970

[CR22] Heinrich B (1979) Majoring and minoring by foraging bumblebees, *Bombus vagance*: an experimental study. Ecology 60:245–255

[CR23] Hills TT (2006) Animal foraging and the evolution of goal-directed cognition. Cogn Sci 30:3–41 21702807 10.1207/s15516709cog0000_50

[CR24] Howard SR, Avarguès-Weber A, Garcia J, Dyer AG (2017) Free-flying honeybees extrapolate relational size rules to sort successively visited artificial flowers in a realistic foraging situation. Anim Cogn 20:627–638. 10.1007/s10071-017-1086-6 28374206 10.1007/s10071-017-1086-6

[CR25] Howard SR, Avarguès-Weber A, Garcia JE, Greentree AD, Dyer AG (2018) Numerical ordering of zero in honey bees. Science 360:1124–1126. 10.1126/science.aar4975 29880690 10.1126/science.aar4975

[CR26] Hribar A, Haun DBM, Call J (2011) Great apes’ strategies to map spatial relations. Anim Cogn 14:511–523 21359655 10.1007/s10071-011-0385-6

[CR27] Hribar A, Haun DBM, Call J (2012) Children’s reasoning about spatial relational similarity: the effect of alignment and relational complexity. J Exp Child Psychol 111:490–500 22154959 10.1016/j.jecp.2011.11.004

[CR28] Jones JE, Antoniadis E, Shettleworth SJ, Kamil AC (2002) A comparative study of geometric rule learning by nutcrackers (*Nucifraga columbiana*), pigeons (*Columba livia*), and jackdaws (*Corvus monedula*). J Comp Psychol 116:350–356 12539930 10.1037/0735-7036.116.4.350

[CR29] Leech R, Mareschal D, Cooper RP (2008) Analogy as relational priming: a developmental and computational perspective on the origins of a complex cognitive skill. Behav Brain Sci 31:357–378 18662435 10.1017/S0140525X08004469

[CR30] Martin-Ordas G (2022) Spontaneous relational and object similarity in wild bumblebees. Biol Lett 18:20220253. 10.1098/rsbl.2022.0253 36043304 10.1098/rsbl.2022.0253 PMC9428533

[CR31] Martin-Ordas G (2023) Relational reasoning in wild bumblebees revisited: the role of distance. Sci Rep 13:22311. 10.1038/s41598-023-49840-5 38102236 10.1038/s41598-023-49840-5 PMC10724225

[CR32] Menzel R, Greggers U, Smith A, Berger S, Brandt R, Brunke S, Bundrock G, Hülse S, Plümpe T, Schaupp F, Schüttler E, Stach S, Stindt J, Stollhoff N, Watzl S (2005) Honey bees navigate according to a map-like spatial memory. Proc Natl Acad Sci U S A 102(8):3040–3045. 10.1073/pnas.0408550102 15710880 10.1073/pnas.0408550102 PMC549458

[CR33] Muth F, Cooper TR, Bonilla RF, Leonard AS (2017) A novel protocol for studying bee cognition in the wild. Methods Ecol Evol 9:78–87

[CR34] Ohashi K, Leslie A, Thomson JD (2008) Trapline foraging by bumble bees: v. effects of experience and priority on competitive performance. Behav Ecol 19:936–948

[CR35] Penn DC, Holyoak KJ, Povinelli DJ (2008) Darwin’s mistake: explaining the discontinuity between human and nonhuman minds. Behav Brain Sci 31:109–130 18479531 10.1017/S0140525X08003543

[CR36] Poti P, Kanngiesser P, Saporiti M, Amiconi A, Blasing B, Call J (2010) Searching in the middle—capuchins’ (*Cebus apella*) and bonobos’ (*Pan paniscus*) behavior during a spatial search task. J Exp Psychol Anim Behav Process 36:92–109 20141320 10.1037/a0015970

[CR37] Quinn PC, Adams A, Kennedy E, Shettler L, Wasnik A (2003) Development of an abstract category representation for the spatial relation between in 6- to 10-month-old infants. Dev Psychol 39:151–163 12518816 10.1037//0012-1649.39.1.151

[CR38] Simms NK, Gentner D (2019) Finding the middle: spatial language and spatial reasoning. Cog Dev 50:177–194. 10.1016/j.cogdev.2019.04.002

[CR40] Smirnova A, Zorina Z, Obozova T, Wasserman E (2015) Crows spontaneously exhibit analogical reasoning. Curr Biol 25:256–260 25532894 10.1016/j.cub.2014.11.063

[CR41] Sovrano VA, Bisazza A, Vallortigara G (2007) How fish do geometry in large and in small spaces. Anim Cogn 10:47–54 16794851 10.1007/s10071-006-0029-4

[CR42] Uttal DH, Sandstrom LB, Newcombe NS (2006) One hidden object, two spatial codes: young children’s use of relational and vector coding. J Cogn Dev 7:503–525

[CR43] Wasserman E, Castro L, Fagot J (2017) Relational thinking in animals and humans: from percepts to concepts. In:J Call, GM Burghardt, IM Pepperberg, CT Snowdon, T Zentall (Eds), *APA *handbook of comparative psychology: Perception, learning, and cognition, American Psychological Association, (pp. 359–384). 10.1037/0000012-017

[CR44] Webb B (2019) The internal maps of insects. J Exp Biol 222(Suppl_1):jeb188094. 10.1242/jeb.188094 30728234 10.1242/jeb.188094

[CR45] R Core Team (2021) R: A language and environment for statistical computing. R Foundation for Statistical Computing, Vienna, Austria. URL https://www.R-project.org/

