# Supplementary material for: Relational similarity in wild bumblebees: the role of spatial alignment complexity
Source: Anim Cogn. 2025 Nov 17;28(1):94. doi: 10.1007/s10071-025-02012-6 (PMC12628461; doi:10.1007/s10071-025-02012-6)
Supplement: Supplementary file 3 — Supplementary information (462 KB) [file 10071_2025_2012_MOESM3_ESM.docx]

**Supplementary information**

**Methods**

**Setup of the Experiments**

**Figure S1**. T-shaped platform and experimental setup used for the present experiments. The Figure represents the *One-Line* condition.

**Results**

**Experiment 1: Parallel vs One-line arrangement**

*Analyses*

A second model was run to analyse whether species had any effect in bees’ performance. *Bombus hypnorum* were not included since the only 2 bees were in the *One-line* condition. Bee’s choice was included as a dependent variable, condition, species and location of the rewarded strip as independent variables and individual bees as random factor. The results showed that neither condition (estimate *SD* = 0.142, *z* = 0.546, *P* = 0.585, 95% CI = -0.387 to 0.682) nor species had any effect on bees’ choices (estimate *SD* = -0.1519, *z* = -0.956, *P* = 0.339, 95% CI = -0.479 to 0.173). The reward location still was significant (middle vs left: estimate *SD* = 1.017, *z* = 3.571, *P* < 0.001, 95% CI = 0.466 to 1.585; middle vs right: estimate *SD* = 1.618, *z* = 5.212, *P* < 0.001, 95% CI = 1.024 to 2.245).

A final model was run including bee’s choice as dependent variable, condition, trial number and location of the rewarded strip as independent variables and individual bees as random factor, the results showed that neither condition (estimate *SD* = 0.063, *z* = 0.252, *P* = 0.019, 95% CI = -0.449 to 0.585) nor trial number had any effect on bees’ choices (estimate *SD* = 0.310, *z* = 0.567, *P* = 0.57, 95% CI = -0.047 to 0.087). The reward location still was significant (middle vs left: estimate *SD* = 0.987, *z* = 3.585, *P* < 0.001, 95% CI = 0.453 to 1.535; middle vs right: estimate *SD* = 1.552, *z* = 5.204, *P* < 0.001, 95% CI = 0.981 to 2.154).

**Experiment 2: Misaligned stimuli arrangement**

*Analyses*

A second model was run to analyse whether species had any effect in bees’ performance. Neither *Bombus hypnorum* nor *Bombus pratorum* were not included since the only 2 bees of each species and they were different spatial alignments. Bee’s choice was included as a dependent variable, condition, species and location of the rewarded strip as independent variables and individual bees as random factor. The results showed that neither condition (estimate *SD* = 0.374, *z* = 1.534, *P* = 0.125, 95% CI = -0.102 to 0.857) nor species had any effect on bees’ choices (estimate *SD* = 0.062, *z* = 0.513, *P* = 0.608, 95% CI = -0.176 to 0.303). The reward location still was significant (B1 vs B2: estimate *SD* = -1.301, *z* = -4.299, *P* < 0.001, 95% CI = -1.905 to -0.716; B1 vs B3: estimate *SD* = -1.107, *z* = -3.709, *P* < 0.001, 95% CI = -1.702 to -0.529). No differences were found between B2 and B3 (estimate *SD* = 0.193, *z* = 0.676, *P* = 0.499, 95% CI = -0.367 to 0.758).

A final model was run including bee’s choice as dependent variable, condition, trial number and location of the rewarded strip as independent variables and individual bees as random factor, the results showed no effect of condition in bees’ performance (estimate *SD* = 0.336, *z* = 1.506, *P* = 0.132, 95% CI = -0.100 to 0.777) or trial (estimate *SD* = 0.002, *z* = 0.068, *P* = 0.945, 95% CI = -0.062 to 0.066). However, there was a significant effect of the rewarded position on bees’ choices. Bees performed better when the reward was in the B1 strip compared to the B2 (estimate *SD* = -1.222, *z* = -4.355, *P* < 0.001, 95% CI = -1.781 to -0.679; Figure 4) and B3 strip (estimate *SD* = -1.160, *z* = -4.169, *P* < 0.001, 95% CI = -0.715 to -0.621; Figure 4). No differences were found between B2 and B3 (estimate *SD* = 0.073, *z* = 0.232, *P* = 0.817, 95% CI = 0.580 to 1.99).
